# The Evaluation of Rac1 Signaling as a Potential Therapeutic Target of Alzheimer’s Disease

**DOI:** 10.3390/ijms241511880

**Published:** 2023-07-25

**Authors:** Huanhuan Wang, Yukie Yamahashi, Marcel Riedl, Mutsuki Amano, Kozo Kaibuchi

**Affiliations:** 1 Department of Cell Pharmacology, Graduate School of Medicine, Nagoya University, 65 Tsurumai, Nagoya 466-8550, Japan; wang.huanhuan@fujita-hu.ac.jp (H.W.); m-amano@med.nagoya-u.ac.jp (M.A.); 2 Division of Cell Biology, International Center for Brain Science, Fujita Health University, Toyoake 470-1192, Japan; yukie.yamahashi@fujita-hu.ac.jp (Y.Y.); 81021040@fujita-hu.ac.jp (M.R.)

**Keywords:** Rac1, Rac GTPase-activating protein, Bcr, p21-activating kinase, nucleus accumbens, aversive learning, Alzheimer disease, microRNA mimic-expressing adeno-associated virus

## Abstract

The Small GTPase Rac1 is critical for various fundamental cellular processes, including cognitive functions. The cyclical activation and inactivation of Rac1, mediated by Rac guanine nucleotide exchange factors (RacGEFs) and Rac GTPase-activating proteins (RacGAPs), respectively, are essential for activating intracellular signaling pathways and controlling cellular processes. We have recently shown that the Alzheimer’s disease (AD) therapeutic drug donepezil activates the Rac1-PAK pathway in the nucleus accumbens (NAc) for enhanced aversive learning. Also, PAK activation itself in the NAc enhances aversive learning. As aversive learning allows short-term preliminary AD drug screening, here we tested whether sustained Rac1 activation by RacGAP inhibition can be used as an AD therapeutic strategy for improving AD-learning deficits based on aversive learning. We found that the RacGAP domain of breakpoint cluster region protein (Bcr) (Bcr-GAP) efficiently inhibited Rac1 activity in a membrane ruffling assay. We also found that, in striatal/accumbal primary neurons, Bcr knockdown by microRNA mimic-expressing adeno-associated virus (AAV-miRNA mimic) activated Rac1-PAK signaling, while Bcr-GAP-expressing AAV inactivated it. Furthermore, conditional knockdown of Bcr in the NAc of wild-type adult mice enhanced aversive learning, while Bcr-GAP expression in the NAc inhibited it. The findings indicate that Rac1 activation by RacGAP inhibition enhances aversive learning, implying the AD therapeutic potential of Rac1 signaling.

## 1. Introduction

Rac1, a member of the Rho-family GTPases, is an intracellular transducer known to control a wide variety of signaling pathways that are critical for cellular processes, including actin reorganization, gene expression, cell viability, and cognitive functions [1,2,3,4]. Emerging evidence indicates that dysfunctional Rac1 signaling is associated with Alzheimer’s disease (AD) [5,6]. Rac1 cycles between an inactive GDP-bound form and an activated GTP-bound form in response to extracellular stimuli, including G protein-coupled receptors (GPCRs) and growth factor receptors [7,8]. The Rac1 activation cycle is controlled by guanine nucleotide exchange factors (GEFs) and GTPase-activating proteins (GAPs): GEFs activate Rac1 by stimulating GDP/GTP exchange, while GAPs inactivate Rac1 by stimulating its GTP hydrolysis reaction [9]. Activated Rac1 interacts with a variety of downstream effectors, including WAVE protein [10], mixed lineage kinase 2 (MLK2) [10], p21-activating kinase (PAK) [10,11,12], and phosphoinositide 3-kinase (PI3K) [13,14]. Among its downstream effectors, PAK is the most characterized [2].

Recently, we have shown that the AD therapeutic drug donepezil, which increases brain acetylcholine levels, activates Rac1-PAK signaling in the striatum/nucleus accumbens (NAc), a mediator of emotional learning, for enhanced aversive learning in wild-type adult mice via the muscarinic acetylcholine receptor 1 (M1R)-PKC cascade [15]. Furthermore, the expression of the constitutively active PAK in NAc enhances aversive learning, indicating that Rac1-PAK signaling activation in NAc is sufficient for enhanced aversive learning. The above findings imply that Rac1 signaling is a potential AD therapeutic target for improving AD-associated learning deficits. In fact, aversive learning is used for preliminary AD drug screening because it allows short-term assessment of the effect of drugs against learning deficits caused by brain cholinergic dysfunction, which mimics AD-associated learning deficits [16]. To facilitate the development of AD therapeutic strategies, it is necessary to find an efficient way to activate Rac1 signaling. Considering that Rac1 activation by RacGEF is transient and that therapeutic drugs usually rely on inhibitors rather than activators, RacGAP inhibition can be a strategy for efficient Rac1 signaling activation. In this study, we tested whether Rac1 activation by RacGAP inhibition in the NAc enhances aversive learning to evaluate Rac1 signaling as a potential AD therapeutic target.

Here, we examined whether Rac1 activation by RacGAP inactivation affected aversive learning and found that conditional knockdown of breakpoint cluster region protein (Bcr), Rac1 GAP, in the NAc enhanced aversive learning. These findings indicate that Rac1-PAK signaling activation by RacGAP inhibition in the NAc enhances aversive learning.

## 2. Results

### 2.1. RacGAP Bcr Knockdown in Striatal/Accumbal Neurons Enhances Rac1-PAK Signaling

To date, 70 RhoGAPs have been identified in humans [17]. RhoGAPs reported to: (1) show single or broad substrate specificity for Rac1, (2) be expressed in the striatum/NAc at the mRNA level, based on Brain Atlas (https://mouse.brain-map.org (accessed on 5 October 2020)) and Pax-db databases (https://pax-db.org (accessed on 5 October 2020)), and (3) be involved in spine morphogenesis, were considered RacGAP candidates. Six RacGAPs, Abr, Bcr, Chimaerin, RICH2, SRGAP2, and SRGAP3, satisfy the criteria [17,18,19,20,21,22]. However, Chimaerin was omitted because its knockout during adulthood did not alter learning and memory [23]. Therefore, SRGAP2, SRGAP3, RICH2, Abr, and Bcr were considered RacGAP candidates for further analysis.

**Figure 1:**
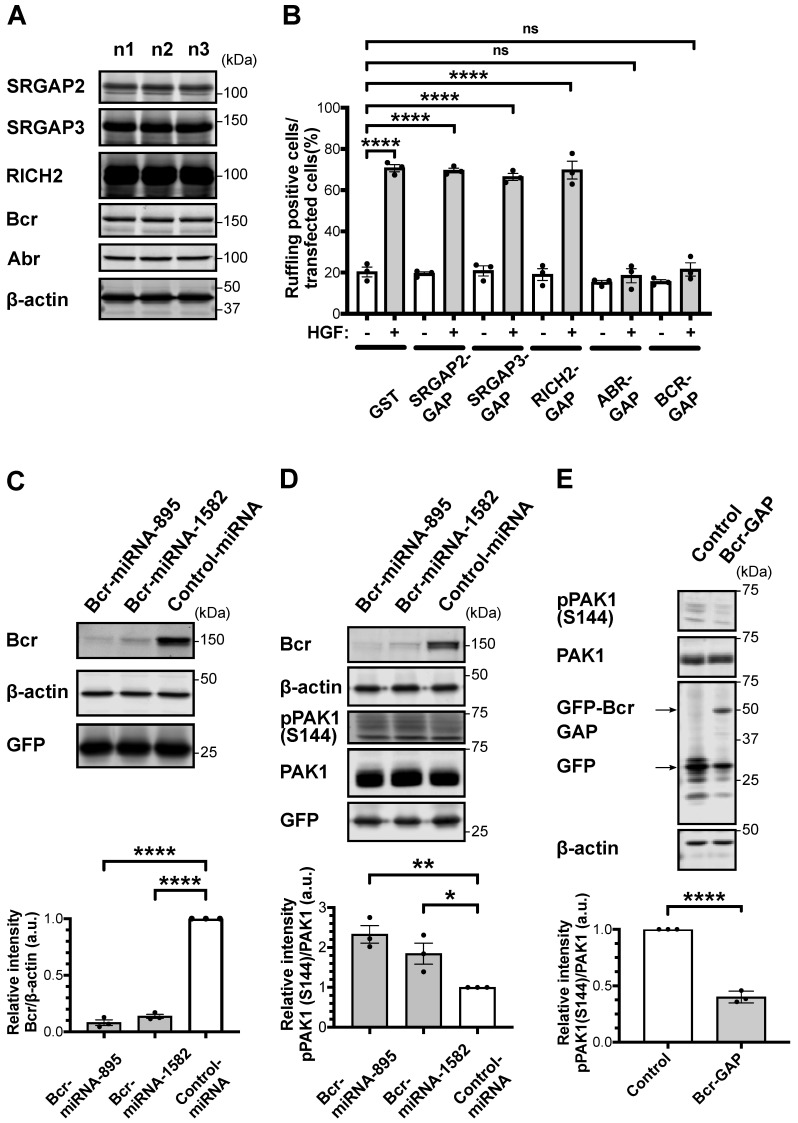
RacGAP Bcr knockdown in striatal/accumbal neurons enhances Rac/PAK signaling. (**A**) Striatal/accumbal brain slices were prepared from three 8-week-old C57BL/6J mice. The panels show the immunoblots of SRGAP2, SRGAP3, RICH, Bcr, Abr, and β-Actin. (**B**) MDCKII cells were transfected with plasmids expressing the GST-tagged RacGAP domain of SRGAP2, SRGAP3, RICH2, Abr, or Bcr and were then stimulated with rHGF (100 pM, 15 min) at 37 °C. Transfected cells showing membrane ruffling were counted under confocal microscope. About 100 transfected cells for each group were counted. The error bars represent the mean ± SEM of three independent experiments. One-way ANOVA followed by Dunnett’s test, ns not significant, **** *p* < 0.0001. (**C**) Primary striatal/accumbal neurons were infected with EmGFP-control miRNA mimic-expressing AAV or EmGFP-Bcr miRNA mimic-expressing AAV at DIV 6. Bcr expression level was quantified by immunoblotting over three independent experiments. Upper panels show representative immunoblots. Quantification of the immunoblotting assay is shown in the bottom panels. The error bars represent the mean ± SEM of three independent experiments. One-way ANOVA followed by Dunnett’s test, **** *p* < 0.0001. (**D**) Primary striatal/accumbal neurons were infected with EmGFP-control miRNA mimic-expressing AAV or EmGFP-Bcr miRNA mimic-expressing AAV at DIV 6. PAK autophosphorylation was quantified by immunoblotting over three independent experiments. Upper panels show representative immunoblots. Quantification of the immunoblotting assay is shown in the bottom panel. The error bars represent the mean ± SEM of three independent experiments. One-way ANOVA followed by Dunnett’s test, * *p* < 0.05, ** *p* < 0.01. (**E**) Primary striatal/accumbal neurons were infected with EGFP-expressing AAV or EGFP-Bcr-GAP-expressing AAV. PAK autophosphorylation was quantified by immunoblotting over three independent experiments. Upper panels show representative immunoblots. Quantification of the immunoblotting assay is shown in the bottom panels. Student’s *t*-test, **** *p* < 0.0001.

To examine the protein expression of the five RacGAP candidates in the striatum/NAc of adult C57BL/6J mice (7–8 weeks old), ex vivo striatal/accumbal brain slices were prepared for subsequent immunoblotting analysis. Immunoblotting analysis of the brain slice lysates showed that all five RacGAP candidates were detected in adult mouse striatum/NAc at the protein level (Figure 1A).

As not all RacGAPs show single specificity for Rac1 [17,24], we examined which RacGAPs efficiently inhibit Rac1 activity. Activated Rac1 is known to mediate membrane ruffling upon extracellular growth factor stimulation [25,26], and historically, growth factor-induced membrane ruffling has been used as an output to examine specificities for RhoGAPs in cells [27]. We therefore performed an HGF-induced membrane ruffling assay to screen for RacGAP candidates that efficiently inhibit Rac1 activity. To this end, MDCKⅡ cells were transfected with plasmids expressing the RacGAP domain of each RacGAP candidate and were then stimulated with 100 pM of recombinant HGF (rHGF). Fifteen minutes after rHGF stimulation, the immunofluorescence analysis was performed, and the number of Rac GAP domain-expressing cells that showed membrane ruffling was counted. The expression of the ABR RacGAP domain (ABR-GAP) and the BCR RacGAP domain (BCR-GAP) significantly decreased the HGF-induced membrane ruffling to the same extent with non-stimulated MDCK II cells, whereas the expression of the SRGAP2 RacGAP domain (SRGAP2-GAP), SRGAP3 RacGAP domain (SRGAP3-GAP), or RICH2 RacGAP domain (RICH2-GAP) did not (Figure 1B and Figure S1). These results indicate that BCR-GAP and ABR-GAP efficiently inhibit Rac1 activity in MDCKⅡ cells. Therefore, we focused on Bcr and Abr for further analysis.

Bcr and Abr are large proteins that contain several different functional domains, including the RacGAP domain. Their RacGAP domains are well conserved, showing a high homology of 81% [28]. Bcr or Abr knockout has been shown to activate Rac1-PAK signaling in the whole brain in vivo, based on the increased PAK autophosphorylation [18]. They have also been shown to enhance spine formation in vivo, a critical factor for learning and memory [18]. Therefore, we investigated whether Bcr or Abr knockdown activates Rac1-PAK signaling in striatal/accumbal neurons. To this end, we utilized the engineered microRNA (miRNA) mimic system, in which a chemically synthesized double-stranded RNA molecule imitates the structure of endogenous miRNA (i.e., miR-155) and cleaves the open reading frame of complementary mRNA (for details, see Material and Methods). Two Bcr miRNA mimic sequences and two Abr miRNA mimic sequences were constructed into adeno-associated virus (AAV) plasmids. AAV-Bcr miRNA mimics or AAV-Abr miRNA mimics were transduced into primary striatal/accumbal neurons for immunoblotting analysis. Both Bcr miRNA mimics knocked down about 90% of the endogenous Bcr protein (Figure 1C). Both Abr miRNA mimics also knocked down endogenous Abr protein (Figure S2). However, their transduction caused cell death, despite the fact that neurons transduced with AAV-negative control or AAV-Bcr miRNA mimics did not when cultured under the same conditions. Thus, we hereafter focused on the effects of AAV-Bcr miRNA mimics.

We next examined whether Bcr knockdown activates Rac1-PAK signaling in primary striatal/accumbal neurons. Rac1-PAK signaling activation was monitored based on PAK1 autophosphorylation level at S144, the site known to disable PAK homodimerization mediated by autoinhibitory domain-kinase domain interaction for PAK activation [29,30,31]. Immunoblotting analysis showed that both Bcr miRNAs mimic increased PAK1 autophosphorylation at S144 in primary striatal/accumbal neurons (Figure 1D), indicating that Bcr knockdown activates Rac1-PAK signaling in striatal/accumbal neurons.

To further confirm that Rac1-PAK activation by Bcr knockdown depends on its RacGAP activity, we transduced AAV-Flex-EGFP-Bcr-GAP and AAV-CaMKII-Cre into primary striatal/accumbal neurons. Immunoblotting analysis showed that Bcr-GAP domain expression significantly decreased PAK1 autophosphorylation at S144 (Figure 1E). Collectively, the above results indicate that the Bcr RacGAP domain inhibits Rac1-PAK signaling in striatal/accumbal neurons and that Rac1-PAK activation by Bcr knockdown depends on its RacGAP activity.

### 2.2. Bcr Knockdown in the NAc Enhances Aversive Learning

**Figure 2:**
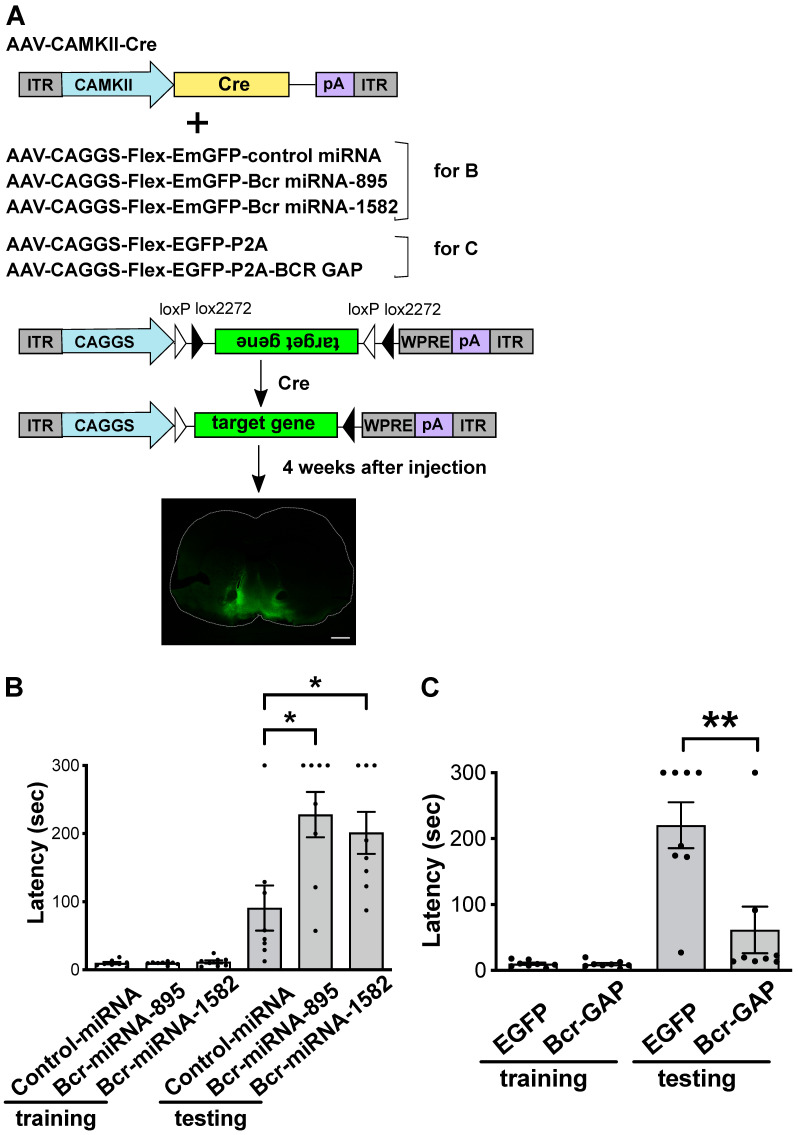
Bcr knockdown in the NAc enhances aversive learning. (**A**) The scheme of Cre-flex system. Adult C57BL/6J mice (7–8 weeks old) were injected into the NAc with AAV-CAMKII-Cre, together with the indicated AAV vectors. Bottom panel shows a representative coronal brain slice showing the expression of AAV-CAGGS-Flex-EGFP-P2A 4 weeks after the injection. Scale bar, 1 mm. (**B**) Adult C57BL/6J mice (7–8 weeks old) were co-injected with AAV-CAMKII-Cre and AAV-CAGGS-Flex-EmGPF-Bcr miRNA mimics into the NAc and were subjected to passive avoidance test 4 weeks after the injection. AAV-mediated expression of Bcr miRNA mimics in the NAc increased the step-through latency 24 h after the mice received electric foot shock (0.47 mA, 4 s) during the passive avoidance test. Data are represented as the mean ± SEM (n = 8, where n is the number of mice for each group), One-way ANOVA followed by Dunnett’s test, * *p* < 0.05. (**C**) Adult C57BL/6J mice (7–8 weeks old) were co-injected with AAV-CAMKII-Cre and AAV-CAGGS-Flex-EGPF-Bcr-GAP into the NAc and were subjected to passive avoidance test 4 weeks after the injection.AAV-mediated expression of Bcr-GAP in the NAc decreased the step-through latency 24 h after the mice received electric foot shock (0.8 mA, 5 s) during the passive avoidance test. Data are represented as the mean ± SEM (n = 8, where n is the number of mice for each group), Student’s *t* test, ** *p* < 0.01.

To investigate the effect of Bcr knockdown on aversive learning, we used the Cre-Flex system to conditionally knockdown Bcr in accumbal neurons. In this system, AAV-CAGGS-Flex-EmGFP-Bcr miRNA mimic and AAV-CAMKII-Cre were co-injected into the NAc of adult C57BL/6J mice (7–8 weeks old), in which Cre recombinase inverts the Bcr miRNA mimic gene to the correct direction specifically in the NAc (Figure 2A). In this system, Bcr miRNA mimics significantly knocked down Bcr expression in the NAc in vivo (Figure S3).

Four weeks later, the injected mice were subjected to a passive avoidance test. In this test, mice were exposed to a mild electric foot shock (0.47 mA, 4 s) as an aversive stimulus in the dark component, where they prefer to stay in nature. Twenty-four hours later, step-through latency from the bright component to the dark component was measured. As a result, Bcr knockdown in the NAc significantly extended the step through latency (Figure 2B), indicating that Bcr knockdown enhances aversive learning.

To validate that the enhanced aversive learning by Bcr knockdown depends on its RacGAP activity, adult WT male mice were co-injected with AAV-CAGGS-Flex-EGFP-Bcr-GAP and AAV-CAMKII-Cre into the NAc (Figure 2A). Four weeks later, the AAV-injected mice were subjected to a passive avoidance test. In an experiment in which an electric foot shock (0.8 mA, 5 s) was used as an aversive stimulus 24 h after the shock, Bcr-GAP expression in the NAc significantly shortened the step through latency (Figure 2C), indicating that Rac1-PAK inactivation by the Bcr RacGAP domain in the NAc inhibits aversive learning. Collectively, the above data indicates that the enhanced aversive learning by Bcr knockdown depends on its RacGAP activity.

## 3. Discussion

Dysfunctional Rac1 signaling has been implicated in AD pathogenesis and AD-associated learning deficits [32,33]. We have previously shown that the AD therapeutic drug donepezil activates Rac1-PAK signaling through the M1R-PKC cascade in striatal/accumbal neurons for enhanced aversive learning [15]. In this study, we found that Rac1-PAK signaling activation by RacGAP Bcr knockdown in the NAc during adulthood enhanced aversive learning. This finding implies that Rac1 signaling is an AD therapeutic potential target, as aversive learning is one of the outputs used for preliminary AD drug screening.

The role of RacGAPs in adult cognition has previously been assessed by global knockout during the embryonic stage [18,23]. However, it is unclear if RacGAPs during development or in adulthood are important in adult cognition because Rac1 is involved in both brain development and spine plasticity in adulthood. The present finding underlines the importance of Rac1 signaling activation during adulthood for adult cognition. It further implies the notion that Rac1 activation by RacGAP inhibition can be used as a therapeutic strategy for improving AD-associated learning deficits, considering that the onset of cognitive decline in AD occurs in adulthood.

As mentioned in the introduction, Rac1 signaling mediates various cellular processes through a wide range of downstream effectors. Historically, the characterization of actin reorganization by Rac1-PAK signaling has been well established [34], which has brought some mechanistic insights into AD pathogenesis, especially spine dysfunction [5,6]. Rac1 is also known to activate PI3K-AKT signaling downstream of GPCR [14], and recent findings show that its activation facilitates cell survival in various cell types [35,36,37]. Given that cell death is a major hallmark of AD, future investigations on whether Rac1 activation inhibits cell death in AD model mice would be of interest to further validate the AD therapeutic potential of Rac1 signaling. Recent findings that M1R-specific agonists inhibit cell death in prion-diseased mice and AD model mice [38,39] support this notion. In this context, we are currently investigating whether M1R-specific agonists activate Rac1 signaling.

The present study focused on Rac1 signaling in the NAc and aversive learning. However, AD patients also show declines in hippocampus-dependent learning and memory. We previously showed that donepezil activates PAK in the dorsal hippocampus and that PAK activation is involved in hippocampus-dependent recognition memory and associative learning [15]. Taken together with the finding that M1R-specific agonists facilitate hippocampus-dependent learning and memory [40], our previous findings imply that ACh-M1R-Rac1-PAK signaling is also involved in hippocampus-dependent learning and memory. For a full evaluation of the therapeutic potential of Rac1, further investigation on whether Rac1 signaling activation by RacGAP inhibition in the hippocampus enhances hippocampus-dependent learning and memory is currently underway.

## 4. Materials and Methods

### 4.1. Antibodies

The following antibodies were used for immunoblotting: mouse monoclonal anti-SRGAP2 (G-10) (#sc-398399,1:400, Santa Cruz Biotechnology, Dallas, TX, USA), mouse monoclonal anti-SRGAP3 (E-11) (RRID: AB_11012147, #sc-374503, 1:400, Santa Cruz Biotechnology, Dallas, TX, USA), mouse monoclonal anti-RICH2(A-7) (#sc-390609, 1:400, Santa Cruz Biotechnology, Dallas, TX, USA), mouse monoclonal anti-BCR(7C6) (RRID: AB_626741, #sc-103, 1:400, Santa Cruz Biotechnology, Dallas, TX, USA), mouse monoclonal anti-ABR(24) (RRID: AB_2221350,#sc-135821, 1:400, Santa Cruz Biotechnology, Dallas, TX, USA), mouse monoclonal anti β-Actin (RRID:AB_2858279, #010-27841, 1:2000, Fujifilm Wako, Osaka, Japan); rabbit polyclonal anti-GFP (RRID:AB_591816, #598, 1:2000, MBL International, Woburn, MA, USA), rabbit polyclonal anti-Phospho-PAK1(S144) (RRID: AB_2299279, #2606S, 1:1000, Cell Signaling Technology, Danvers, MA, USA), rabbit polyclonal anti-PAK1 (RRID: AB_330222, #2602S, 1:1000, Thermo Scientific, Waltham, MA, USA), rabbit polyclonal anti-BCR (RRID: AB_2063777, #3902S, 1:1000, Cell Signaling Technology, Danvers, MA, USA) for Figure S3; goat anti-rabbit IgG Alexa Fluor 680 (RRID: AB_2535758, Thermo Scientific, Waltham, MA, USA), anti-Rabbit IgG DyLight 800 conjugate (RRID: AB_10697505, Cell Signaling Technology, Danvers, MA, USA), goat anti-mouse IgG DyLight 800 conjugate (RRID: AB_10693543, Cell Signaling Technology, Danvers, MA, USA), goat anti-mouse IgG Alexa Fluor 680 (RRID: AB_2535724, A21058, Invitrogen, Waltham, MA, USA) secondary antibodies (1: 20,000).

The following antibodies were used for immunohistochemistry: anti-GST (1:1000, Homemade), Alexa Fluor 555-conjugated donkey anti-rabbit IgG (1:600, RRID: AB_162543, #A31572, Invitrogen, Waltham, MA, USA), and Alexa Fluor 488-conjugated Phalloidin (1:50, #A12379, Invitrogen, CA, USA).

### 4.2. Cell Culture and Transfection

MDCK II cells were cultured in DMEM (Dulbecco’s Modified Eagle’s Medium-High Glucose, D5796-500ML, Sigma-Aldrich, St. Lois, MO, USA) containing 10% CS(BOVINE CALF SERUM, #SH30072.03, Thermo Scientific, Waltham, MA, USA) at 37 °C. Cells were transfected by Lipofectamine 2000 (#11668-500, Invitrogen, Waltham, MA, USA) with pEF-BOS-GST, pEF-BOS-GST-SRGAP2-GAP, pEF-BOS-GST-RICH2-GAP, pEF-BOS-GST-Abr-GAP, and pEF-BOS-GST-Bcr-GAP, respectively.

### 4.3. Animal

C57BL/6J (RRID: IMSR_JAX:000664) male mice and pregnant ICR female mice were purchased from Japan SLC (Shizuoka, Japan). Mice were housed at a density of four mice per cage under a standard 12 h light/dark cycle (light phase 8:00–20:00) at a constant temperature of 23 ± 1 °C. Food and water were available throughout the experiments. The male mice used in this study were 7~11 weeks old and had a body weight of 22–30 g. All animal experiments were approved and performed in accordance with the guidelines for the care and use of laboratory animals established by the Animal Experiments Committee of Fujita Health University (reference number: AP20037). All experiments were conducted in compliance with the ARRIVE guidelines.

### 4.4. Plasmid Construction

cDNAs encoding full-length human SRGAP2 (KIAA0456), full-length SRGAP3 (KIAA0411), full-length RICH2 (KIAA0672) were purchased from Kazusa DNA Research Institute (Chiba, Japan), and cDNAs encoding full-length human Bcr (NM_004327.3) and full-length Abr (NM_021962.4) were cloned from human fetal brain cDNA library. GAP domain fragments, SRGAP2-GAP (1483–2385 bp), SRGAP3-GAP (1027–2094 bp), RICH2-GAP (778–1302 bp), Abr-GAP (1908–2580 bp), and Bcr-GAP (3150–3816 bp), were obtained by PCR amplification from the above cDNAs. The fragments were then cloned into pEF-BOS-GST expression vector.

Two Bcr engineered miRNA mimic oligos (NM001081412.2, miRNA-Bcr-895: ^895^CTTCCTGAAGGACAACCTAAT^915^; miRNA-Bcr-1582: ^1582^CAAAGTGAGTGAGCTGGATCT^1602^) and two Abr engineered miRNA mimic oligos (NM198895.2, miRNA-Abr-1111: ^1111^TCTCCCAGAATTTCCTGTCTA^1131^; miRNA-Abr-2320: ^2320^TTGAGGAGTTGGGCATCTACA^2340^) targeting the mouse BCR gene or mouse ABR gene were automatically designed by using BLOCK-iT RNAi Designer from Thermo Fisher SCIENTIFIC website (https://rnaidesigner.thermofisher.com/rnaiexpress/ (accessed on 4 December 2020)). The superscript numbers represent the first and last nucleotides of the target gene. As negative control, miRNA mimic (GTCTCCACGCGCAGTACATTT) that is predicted not to target any known vertebrate gene was used and purchased from Thermo Fisher SCIENTIFIC. The DNA oligos were annealed to generate double-stranded oligos in accordance with BLOCK-iT^TM^ Pol II miR RNAi Expression Vector Kit user manual. The double-stranded oligos were cloned into pcDNA6.2-GW: EmGFP-miR vector, in which miRNA sequence derived from endogenous miR-155 sequence is inserted into the 3′-UTR of the EmGFP gene at BsaⅠrestriction enzyme site. The double-stranded oligos accompanied by the miRNA sequence were subcloned into the multiple cloning site (MCS) of the pAAV-CAGGS-Flex-EmGFP-miR-neg and pAAV-CAGGS-EmGFP-miR-neg at BamHⅠand XhoⅠrestriction enzyme site to allow AAV-mediated expression of Bcr miRNA mimics or Abr miRNA mimics.

### 4.5. Primary Neuron Culture

Primary striatal/accumbal neurons from E16 mouse embryos of ICR female mice were dissected on a 35 mm Petri dish on ice under a Leica stereo microscope as previously described with some modifications [41]. The dissected striatal/accumbal tissues were digested into single cells by utilizing Neuron Dissociation Solutions (Code No. 291-78001, FUJIFILM Wako, Osaka, Japan). Striatal/accumbal neurons were plated onto 10 µg/mL Poly-D-lysine hydrobromide (PDL) (P6407-5MG, Sigma-Aldrich, St. Lois, MO, USA) coated 6-well plate at a density of 1 × 10^6^ cells/well with Neurobasal Plus medium (REFA3582901, Gibco, NY, USA) containing 10% FBS (Fetal Bovine Serum, #173012, Sigma-Aldrich, St. Lois, MO, USA). 2 h later, medium was changed to Neurobasal Plus medium containing B27 Plus Supplement (50×) (20 mL/L) (REFA3582801, Gibco, NY, USA). At DIV 3, half of the medium was aspirated and replaced with the same volume of fresh medium containing 2 uM Arac(#C-6645-25MG, Sigma-Aldrich, St. Lois, MO, USA) to reduce the number of nonneural cells (1 µM final dilution). AAV-miRNA mimics were transduced (MOI:10000) into striatal/accumbal neurons at DIV 6. Seventy-two hours after AAV transduction, half of the medium was aspirated and replaced with the same volume of fresh medium. The medium replacement was repeated every 2–3 days.

### 4.6. Cell Ruffling Assay

MDCK II cells were seeded at about 1.0 × 10^4^ cells/well into 12 well plate containing PDL-coated cover glass and cultured in DMEM (Dulbecco’s Modified Eagle’s Medium-High Glucose, D5796-500ML, Sigma-Aldrich, St. Lois, MO, USA) containing 10% CS (BOVINE CALF SERUM, #SH30072.03, Thermo Scientific, Waltham, MA, USA) at 37 °C. Cells were transfected with the indicated vectors. Twenty-four hours after transfection, medium was changed to DMEM medium without serum, and cells were incubated at 37 °C for another 24 h. Forty-eight hours after transfection, cells were stimulated with 100 pM recombinant HGF (#082-09321, Fujifilm Wako, Osaka, Japan) for 15 min at 37 °C for subsequent immunohistochemical analysis. About 100 transfected cells at the outer edge of the colony were observed under confocal microscope (LSM780, Carl Zeiss, Jena, Germany). Among them, the number of transfected cells that show membrane ruffling was counted.

### 4.7. Ex Vivo Sample Preparation

Striatal/accumbal slices for immunoblotting analysis were prepared as previously described [15]. Male C57BL/6J mice (7–8 weeks old) were decapitated for brain removal. Coronal brain slices (thickness: 350 μm) were prepared using VT1200S vibratome (Leica Microsystems, Wetzlar, Germany) in cold oxygenated Krebs buffer (124 mM NaCl, 4 mM KCl, 26 mM NaHCO_3_, 1.5 mM CaCl_2_, 1.25 mM KH_2_PO_4_, 1.5 mM MgSO_4_, and 10 mM D-glucose, pH 7.4). Striatum/NAc were dissected under stereomicroscopy (Leica Microsystems) and snap frozen in liquid nitrogen and stored at −80 °C until assayed. The slices were sonicated in lysis buffer (1% SDS, 1 mM EDTA, 1 mM DTT, 1% glycerol, 50 mM Na_2_HPO_3_, phosphatase inhibitor calyculin A (50 nM) (Fujifilm Wako), protease inhibitor cocktail (Roche), pH 7.0) for 20 s. Lysates were immediately heated at 70 °C for 10 min and briefly centrifuged at 15,000 rpm for 1 min to remove debris. The protein concentration of lysates was determined by BCA assay (Fujifilm Wako, Osaka, Japan).

### 4.8. In Vivo Sample Preparation

In vivo sample preparation for immunoblot analysis was performed as previously described with some modifications [15]. After decapitation, mouse heads were immediately immersed in liquid nitrogen for ~4 s, and the whole brains were removed. The brains were coronally sectioned (thickness: 2 mm) by using a mouse brain slicer matrix (#MBS-S1C, Brain Science Idea Co., Ltd., Osaka, Japan) chilled on ice. NAc was collected using 1.5 mm diameter biopsy punch (Kai corporation, Tokyo, Japan) on ice-cold plate. The tissue was snap-frozen in liquid nitrogen and stored at −80 °C until assayed. The sonication procedure was carried out as described in Section 4.7.

### 4.9. SDS-PAGE Immunoblotting Assay

Samples were loaded onto 7% acrylamide gel or 12% acrylamide gel, and SDS-PAGE electrophoresis was performed. After electrophoresis, proteins in gels were transferred to PDVF membrane (#IPFL00010, Millipore, Cork, Ireland). Membranes were blocked for 30 min with 20% Blocking one (Code 03953-95, Nacalai Tesque, Kyoto, Japan), 0.2% Tween in MQ water, or Blocking-One P (Code 05999-84, Nacalai Tesque, Kyoto, Japan) at room temperature and incubated overnight at 4 °C with the indicated primary antibodies. The membranes were washed with 0.05% Tween/Tris-buffered saline (TBST; 20 mM Tris, 150 mM NaCl, 0.05% Tween 20, pH 7.6). Afterward, the membranes were incubated with secondary antibodies at room temperature (RT) for 30 min and washed three times with TBST. Proteins were detected using an infrared imaging system (LI-COR Biosciences, Lincoln, NE, USA). Band intensities were quantified using Image Studio software (Version 3.1.4, LI-COR Biosciences, Lincoln, NE, USA).

### 4.10. AAV Preparation

AAV vectors were prepared and tittered as described previously, with some slight modifications [15]. Briefly, AAV plasmid, pHelper plasmid (Cell Biolabs, San Diego, CA, USA), and pAAV-DJ plasmid (Cell Biolabs, San Diego, CA, USA) were transfected into HEK293FT cells, and 72 h later, HEK293FT cells were harvested. AAVs were extracted through four freeze-thaw cycles and purified by two Cscl/PBS ultracentrifugations using a SWi40 rotor. After the 2nd round of ultracentrifugation, AAV-rich fraction was collected and dialyzed using a dialysis cassette Slide-A-lyzer (#87730, Thermo Scientific, IL, USA) to remove Cscl. qPCR (#QKD-201, TOYOBO, Osaka, Japan) was performed to estimate final AAV titer.

### 4.11. AAV Injection

AAV injection was performed as described previously [15]. AAV injections were performed on 7–8-week-old C57BL/6J mice with an anesthetic mixture of medetomidine (0.3 mg/kg, i.p.) (Nippon Zenyaku Kogyo, Koriyama, Japan), midazolam (4 mg/kg, i.p.) (Maruishi Pharmaceutical, Osaka, Japan), and butorphanol (5 mg/kg, i.p.) (Meiji Seika Pharma, Tokyo, Japan) and positioned in a stereotaxic frame (David Kopf, Tujunga, CA, USA). AAV-CAGGS-Flex-EmGFP Bcr miRNA mimic and AAV-CAMKII-Cre recombinase (1.0 × 10^12^ genome copies/mL) were coinjected into NAc at a rate of 0.1 µL/min through a glass capillary at a 10° angle (four sites, 0.5 μL/site, bregma: +1.6 mm, ±1.5 mm, −4.4 mm; and +1.0 mm, ±1.6 mm, −4.5 mm). The step-through passive avoidance test was performed four weeks after the injection.

### 4.12. Passive Avoidance Test

Step-through passive avoidance test was performed as previously described with some modifications [15]. Shuttle box for passive avoidance testing (Model ENV-018MD, Med Associates Inc., St. Albans, VT, USA) was used in this study. The apparatus consists of two compartments: a bright compartment and a dark compartment (dimensions of each compartment: W 21.5 cm × D 17.0 cm × H 21.0 cm). The test consists of three phases: habituation phase, training phase, and testing phase. Each phase was carried out 24 h apart. The step-through latency was measured in each phase using MED-PC V software (Version 5.1, Brainscience Idea, Osaka, Japan). On day 1 (habituation phase), the mouse was put in the bright compartment. One minute later, the door opened, and the mouse was given 2 min to enter the dark compartment. On day 2 (training phase), the mouse was put in the bright compartment. One minute later, the mouse was allowed to enter the dark compartment. Once the mouse entered, the door closed automatically. Three seconds later, an electric foot shock (0.47 mA, 4 s or 0.80 mA, 5 s) was applied. The mouse was returned to the home cage 20 s later. On day 3 (testing phase), the mouse was put in the bright compartment, and the door opened 1 min later. The step-through latency was recorded up to 300 s.

### 4.13. Immunohistochemical Analysis

For membrane ruffling assay, MDCKII cells were fixed with 3.7% Formaldehyde/PBS for 10 min after HGF stimulation. Cells were rinsed with 1xPBS three times (10 min per wash) and were then permeabilized with 0.2% Triton X-100/PBS for 10 min at RT. After cells were washed with 1XPBS twice (10 min per wash), they were blocked with 1% BSA/PBS (filtered) for 30 min at RT. Cells were then stained for 1 h at RT or overnight at 4 °C with the indicated primary antibodies diluted with 1%BSA/PBS (filtered). Cells were washed three times with 1xPBS (10 min per wash) and incubated with Alexa Fluor 555-conjugated donkey anti-rabbit IgG secondary antibody for 1 h at RT. F-actin was visualized by staining with Alexa fluor 488-Phalloidin. After three washes with 1xPBS (10 min per wash), coverslips with cells were mounted onto the 76 mm × 26 mm slide glass (#S0318, MATSUNAMI, Osaka, Japan) using Fluoromount (#DBS-K024-25ML, DIAGNOSTIC BIOSYSTEMS INC., Hague, The Netherlands). Image acquisition was performed using a confocal microscope (LSM780, Carl Zeiss, Jena, Germany) with a 63× objective lens (for Figure S1) or 20× objective lens (for Figure 1B) with identical gain and laser settings.

For the AAV expression check experiment, mice were anesthetized with an anesthetic mixture of medetomidine (0.3 mg/kg, i.p.) (Nippon Zenyaku Kogyo, Koriyama, Japan), midazolam (4 mg/kg, i.p.) (Maruishi Pharmaceutical, Osaka, Japan), and butorphanol (5 mg/kg, i.p.) (Meiji Seika Pharma, Tokyo, Japan) for deep and rapid anesthesia, and transcardially perfused with 4% Formaldehyde/PBS, pH 7.4 by using peristaltic pump. A total of 100 mm thick coronal brain slices were prepared using a vibratome (LECIA VT 2000S, Leica Microsystems, Wetzlar, Germany). The entire coronal brain section image was acquired by tile scanning using confocal microscope (LSM780, Carl Zeiss, Jena, Germany) with a 10 × objective lens.

### 4.14. Quantification and Statistical Analysis

Sample size was chosen according to previous studies [42,43]. No animals were excluded from the analyses. Animals were allocated randomly into experimental groups. The investigator was not blinded to group allocation during data collection, as only the investigator, who gave handling habituation, can access mice during behavior tests to avoid any types of stress.

All data are expressed as the mean ± standard error of the mean of at least three independent experiments. The statistical analysis was performed using Prism version 6 software (GraphPad, La Jolla, CA, USA). In all experiments, one-way ANOVA followed by Dunnett’s multiple comparison test or Student’s *t* test (two-tailed) was used. *p* values of <0.05 were considered statistically significant.

## Data Availability

The data presented in this study are available in the article and Supplementary Material.

## Supplementary Materials

The supporting information can be downloaded at: https://www.mdpi.com/article/10.3390/ijms241511880/s1.
