# Supplementary material for: The Evaluation of Rac1 Signaling as a Potential Therapeutic Target of Alzheimer’s Disease
Source: Int J Mol Sci. 2023 Jul 25;24(15):11880. doi: 10.3390/ijms241511880 (PMC10418761; doi:10.3390/ijms241511880)
Supplement: Supplementary file 1 [file ijms-24-11880-s001.zip › 230724 supplementary figure S1.pdf]

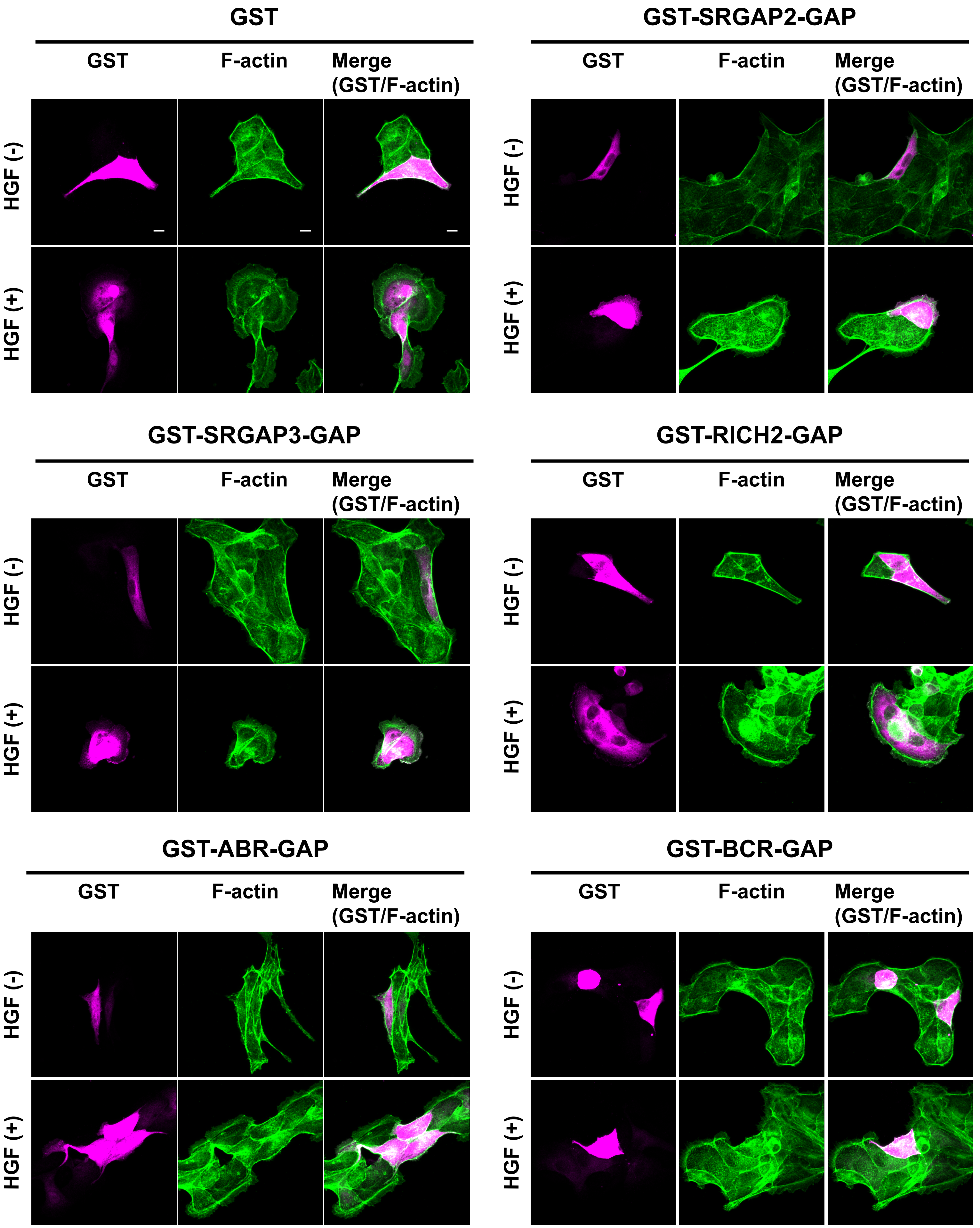

Supplementary figure S1. Membrane ruffling assay. MDCKII cells were transfected with plasmids expressing GST-tagged RacGAP domains of SRGAP2, SRGAP3, RICH2, ABR, or BCR and were then stimulated with
rHGF (100 pM) at 37°C for 15 min. Cells were stained with anti-GST antibody and Alexa Fluor 488-conjugated phalloidin for immunohistological analysis. Scale bar, 10 µm.
