# Supplementary material for: The Evaluation of Rac1 Signaling as a Potential Therapeutic Target of Alzheimer’s Disease
Source: Int J Mol Sci. 2023 Jul 25;24(15):11880. doi: 10.3390/ijms241511880 (PMC10418761; doi:10.3390/ijms241511880)
Supplement: Supplementary file 1 [file ijms-24-11880-s001.zip › 230724 supplementary figure S2.pdf]

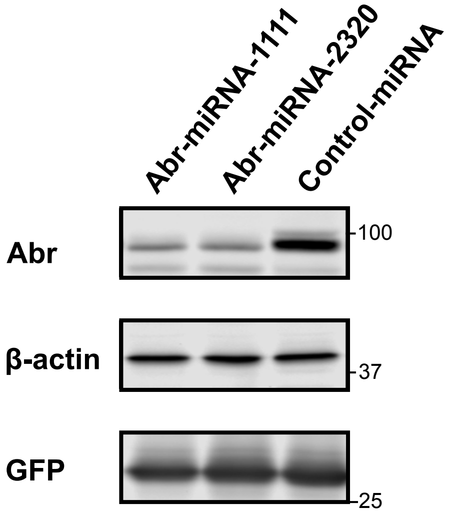

Supplementary figure S2. RacGAP Abr knockdown in striatal neurons. Primary striatal neurons were infected with control miRNA expressing AAV or Abr miRNA expressing AAV at DIV 6. Lysates were immunoblotted with the indicated antibodies.
