# Supplementary material for: The Evaluation of Rac1 Signaling as a Potential Therapeutic Target of Alzheimer’s Disease
Source: Int J Mol Sci. 2023 Jul 25;24(15):11880. doi: 10.3390/ijms241511880 (PMC10418761; doi:10.3390/ijms241511880)
Supplement: Supplementary file 1 [file ijms-24-11880-s001.zip › 230724 supplementary figure S3.pdf]

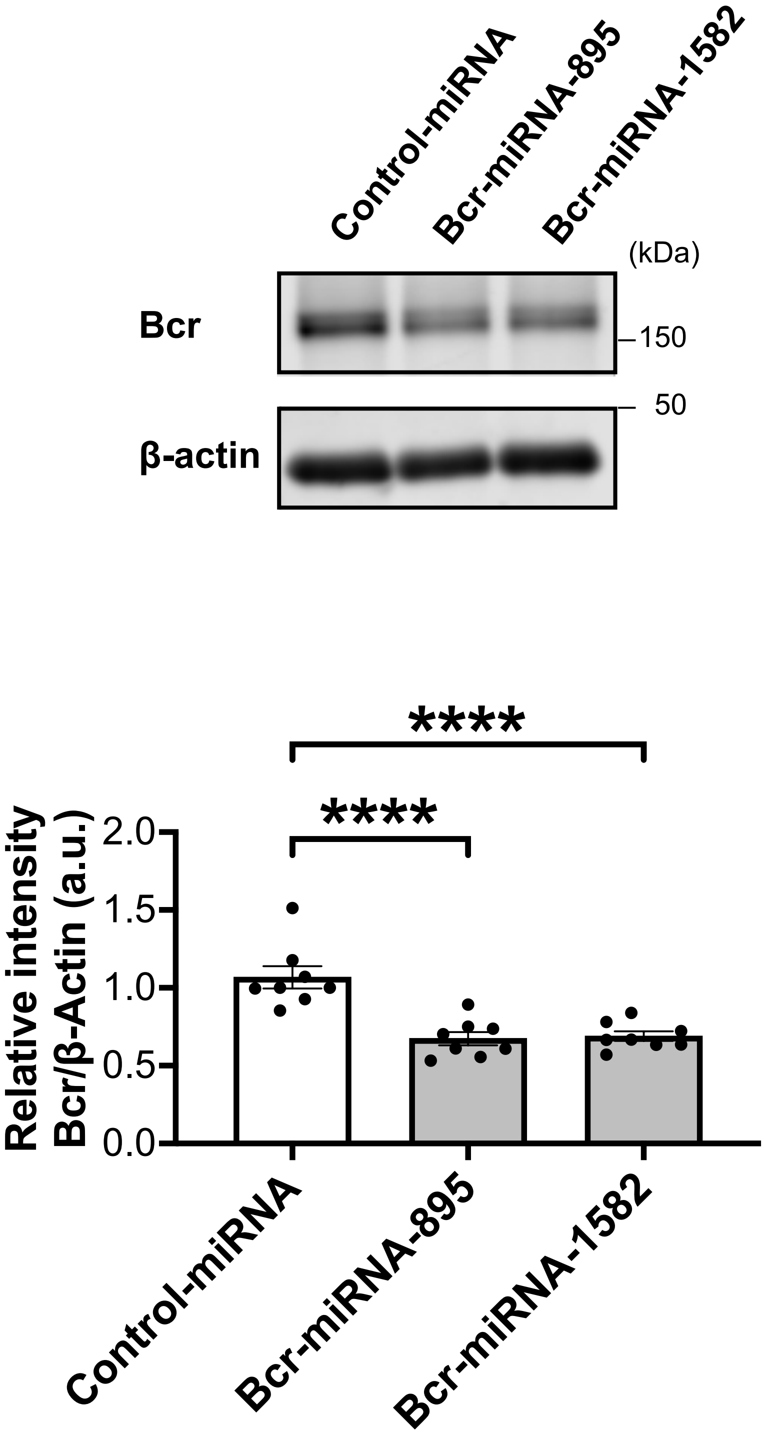

Supplementary figure S3. RacGAP Bcr knockdown in the NAc in vivo. Control miRNA expressing AAV or Bcr miRNA expressing AAV were injected into the NAc of C57BL/6J mice. Four weeks later, Bcr expression in the NAc was quantified by immunoblotting. n=8. Error bars indicate mean ± SEM. One-way ANOVA followed by Dunnett's test, **** $p < 0.0001$.
